# A Novel *WT1* Mutation Identified in a 46,XX Testicular/Ovotesticular DSD Patient Results in the Retention of Intron 9

**DOI:** 10.3390/biology10121248

**Published:** 2021-11-30

**Authors:** Dmytro Sirokha, Olexandra Gorodna, Yakov Vitrenko, Nataliya Zelinska, Rafal Ploski, Serge Nef, Jadwiga Jaruzelska, Kamila Kusz-Zamelczyk, Ludmila Livshits

**Affiliations:** 1 Institute of Molecular Biology and Genetics, National Academy of Sciences of Ukraine, 03143 Kyiv, Ukraine; d.a.sirokha@imbg.org.ua (D.S.); a.v.gorodna@imbg.org.ua (O.G.); 2 IMG—Integrated Medical Group, 6K Mykil’ska-Slobids’ka St., 02002 Kyiv, Ukraine; vitrenko@im-group.com.ua; 3 Ukrainian Scientific and Practical Center for Endocrine Surgery, Transplantation of Endocrine Organs and Tissues, Ministry of Health of Ukraine, 01021 Kyiv, Ukraine; znb@ukr.net; 4 Department of Medical Genetics, Warsaw Medical University, 02-106 Warsaw, Poland; rploski@wp.pl; 5 Department of Genetic Medicine and Development, Faculty of Medicine, University of Geneva, 1211 Geneva, Switzerland; Serge.Nef@unige.ch; 6 Institute of Human Genetics, Polish Academy of Sciences, 60-479 Poznan, Poland; jadwiga.jaruzelska@igcz.poznan.pl

**Keywords:** disorder/difference of sexual development (DSD), testicular DSD (TDSD), ovotesticular DSD (OTDSD), *WT1* gene, Wilms’ tumor 1 protein, splice site mutation, zinc finger, KTS

## Abstract

**Simple Summary:**

Disorders/differences of sexual development are very diverse. Among them is a condition characterized by the presence of testicular tissue in people with female chromosomes, which is typically manifested by male or ambiguous genitalia. While genetic counseling is beneficial for these people and their families, the genetic causes of these cases are only partially understood. We describe a new mutation in the *WT1* gene that results in the presence of testicular tissue in a child with a female karyotype. We propose molecular mechanisms disrupted by this mutation. This finding widened our understanding of processes that govern sexual development and can be used to develop diagnostic tests for disorders/differences of sexual development.

**Abstract:**

The 46,XX testicular DSD (disorder/difference of sexual development) and 46,XX ovotesticular DSD (46,XX TDSD and 46,XX OTDSD) phenotypes are caused by genetic rearrangements or point mutations resulting in imbalance between components of the two antagonistic, pro-testicular and pro-ovarian pathways; however, the genetic causes of 46,XX TDSD/OTDSD are not fully understood, and molecular diagnosis for many patients with the conditions is unavailable. Only recently few mutations in the *WT1* (*WT1* transcription factor; 11p13) gene were described in a group of 46,XX TDSD and 46,XX OTDSD individuals. The *WT1* protein contains a DNA/RNA binding domain consisting of four zinc fingers (ZnF) and a three-amino acid (KTS) motif that is present or absent, as a result of alternative splicing, between ZnF3 and ZnF4 (±KTS isoforms). Here, we present a patient with 46,XX TDSD/OTDSD in whom whole exome sequencing revealed a heterozygous de novo *WT1* c.1437A>G mutation within an alternative donor splice site which is used for −KTS *WT1* isoform formation. So far, no mutation in this splice site has been identified in any patient group. We demonstrated that the mutation results in the retention of intron 9 in the mature mRNA of the 46,XX TDSD/OTDSD patient. In cases when the erroneous mRNA is translated, exclusively the expression of a truncated *WT1* +KTS protein lacking ZnF4 and no −KTS protein occurs from the mutated allele of the patient. We discuss potential mechanisms and pathways which can be disturbed upon two conditions: Absence of Zn4F and altered +KTS/−KTS ratio.

## 1. Introduction

The primary events of gonadal sex determination are genetically controlled in mammals. Distinct pathways lead to the development of testes or ovaries. The pro-testicular pathway is triggered by the *SRY* (sex-determining region Y) gene located on chromosome Yp11.2. In the absence of *SRY*, the pro-ovarian WNT4/RSPO1 (R-spondin 1) and Catenin beta 1 pathways are activated [1,2]. Male and female genetic programs compete and repress each other. An imbalance between the specific components of these antagonistic pathways results in disorders/differences of sex development (DSD).

DSD are congenital conditions in which chromosomal, gonadal or anatomical sex is atypical [3]. The variety of DSD phenotypes reflects a complexity of genetic networks of sex determination and maintenance. Among them there are 46,XX testicular DSD (TDSD) and ovotesticular DSD (OTDSD), two DSD phenotypes distinguished by the presence of testicular tissue despite a female 46,XX karyotype (MIM 400045). Males with 46,XX TDSD typically have small azoospermic testes [4]. In turn, 46,XX OTDSD refers to individuals who have both ovarian and testicular tissue in their gonads. Their genitalia are usually ambiguous, with the degree of masculinization proportional to the amount of testicular tissue [5].

The most common cause of 46,XX TDSD/OTDSD is the translocation of *SRY* to chromosome X. In turn, genetic causes of the *SRY*-negative 46,XX TDSD/OTDSD are variable. Among them, there are chromosomal rearrangements that result in the upregulation of *SRY*-related HMG-box genes *SOX3* (Xq27.1), *SOX9* (17q24.3) and *SOX10* (22q13.1) which are able to trigger testicular differentiation when their levels are sufficiently increased [6,7,8,9]. There are also loss of function mutations of pro-ovarian signalling pathway *RSPO1* (1p34.3) and *WNT4* (1p36.12) genes [10,11]. More recently, several mutations in the zinc finger 4 (ZnF4) domain of the *WT1* gene have been identified in a group of 46,XX TDSD/OTDSD individuals [12,13], but still genetic causes of 46,XX TDSD/OTDSD are not fully understood and molecular diagnosis for many patients with the conditions is unavailable.

The *WT1* gene plays an essential role in the normal development of the urogenital system [14]. Protein isoforms encoded by *WT1* contain a DNA/RNA binding domain consisting of four zinc fingers (ZnF) and a motif of three amino acids (KTS) present or absent between the ZnF3 and the ZnF4 (±KTS isoforms). The presence or absence of KTS is an effect of an alternative splicing which is conserved in all vertebrates, suggesting that +KTS and −KTS are functionally distinct [15]. Indeed, both isoforms differ in affinity to DNA/RNA, with −KTS having a higher affinity to DNA, while +KTS binds preferentially to RNA [16,17]. The +KTS is expected to function in splicing and regulating stability of mRNA [17,18,19]. Isoforms +KTS and −KTS bind to different DNA sequence motifs as well as to different regions of target genes, resulting in a diverse influence on transcription. While the −KTS isoform preferentially binds close to transcription start site as well as to enhancers, thus being related predominately to transcriptional activation, +KTS binds mostly within gene bodies and is related to both activation and repression [20]. Since +KTS and −KTS bind largely to the same genes [20,21] and are able to dimerize [22], it is expected that +KTS and −KTS could cooperate in transcriptional repression.

Distinct roles of each isoform (+KTS and −KTS) in gonadal development were shown in the mouse model. Namely, it was demonstrated that +KTS is essential for testicular determination (it activates *SRY* and *SOX9*), while it represses ovarian development (it downregulates a pro-ovarian *NR0B1*—nuclear receptor subfamily 0 group B member 1 gene; Xp21.2). On the other hand −KTS is needed for both male and female gonadal development [23]. The proper ratio between those two functionally different +KTS and −KTS *WT1* isoforms seems to be critical for correct sexual development since a heterozygous mutation disrupting one alternative splice site and resulting in lower +KTS level causes Frasier syndrome (MIM 136680), characterized among other features by 46,XY gonadal dysgenesis [24,25].

We report a 46,XX TDSD/OTDSD patient who has a novel de novo heterozygous *WT1* gene mutation (*WT1*:c.1437A>G) within an alternative donor splice site. We showed that the mutation results in the retention of intron 9 in the mature mRNA of the patient. Based on the sequence of the aberrant mRNA containing the intron retained, it is to be expected that the mutated allele of the patient expresses exclusively *WT1* +KTS protein lacking ZnF4 and no −KTS isoform, and thus the normal +KTS/−KTS ratio is altered.

## 2. Results

### 2.1. 46,XX TDSD/OTDSD Case Report

The patient was registered as a male at birth. His height and weight were normal. At the age of two months the patient was examined due to hypospadias and bilateral cryptorchidism. At the age of 13 months the genitalia were assessed as stage 2 according to the Prader scale. There was a palpated gonad in the right inguinal canal, while no palpated formation was found in the left one. Ultrasound examination revealed a 16.5 mm × 7.5 mm × 9.5 mm gonad in the right inguinal canal, while neither uterus nor gonad were found in the pelvis. The height was 74 cm (normal for a girl and −1 SD for a boy), weight 9.3 kg, normal BMI 16.9 kg/m^2^. The next examination was conducted at the age of 14 months. Ultrasound investigation revealed the right gonad to be 9 mm × 3 mm and the left one 9 mm × 4 mm in inguinal canals. They could easily move to the abdominal cavity through a wide inguinal ring. Urogenital sinus and fluorocolpos were detected in the projection of the vagina. Magnetic resonance imaging (MRI) of the pelvic organs revealed prostate and phallus in a typical place. A uterus was not detected using both MRI and ultrasound examination. No signs of Wilms’ Tumor or renal anomalies were found in the patient.

Hormonal investigation at the age of two months showed that estradiol (E2) was within the normal range for both sexes, luteinizing hormone (LH) was higher than normal for both sexes and follicle-stimulating hormone (FSH) was within the normal range for girls and higher than the norm for boys. Further hormonal tests at the age of 14 months revealed that anti-Müllerian hormone (AMH) level was higher than normal for girls and normal for boys; dihydrotestosterone (DHT), luteinizing hormone (LH) and Follicle-Stimulating Hormone (FSH) were within the normal range for both sexes. 17-hydroxyprogesterone (17-OHP) was at the upper limit of the normal range. Human chorionic gonadotropin (hCG) testosterone stimulation test showed a high response of testosterone indicating presence of Leydig cells in gonad(s) and proper testosterone biosynthesis (Table 1). Cytogenetic studies of the patient showed a 46,XX karyotype without mosaicism and a lack of chromosome Y-specific sequences including *SRY* gene.

Taking into consideration (1) the male characteristics of sexual development of the patient—absence of uterus, presence of phallus and prostate—(2) the female karyotype, (3) normal level of 17-OHP excluding congenital adrenal hyperplasia, (4) normal-for-boys level of AMH excluding gonadal dysgenesis and (5) the result of the hCG stimulation indicating the presence of testicular tissue, we concluded that the patient suffered from 46,XX TDSD. However 46,XX OTDSD cannot be ruled out because the patient’s gonadal tissue was not available for the study, and without histopathological examination, it is impossible to distinguish between the two conditions. Therefore the temporal diagnosis was 46,XX TDSD or 46,XX OTDSD (46,XX TDSD/OTDSD).

### 2.2. Identification of the Heterozygous WT1:c.1437A>G Mutation in the Patient

We performed whole exome sequencing (WES) of the patient and his parents to search for the genetic determinants of 46,XX TDSD/OTDSD. The sequencing manifested the mean coverage > 145; 95.3% of the targets were covered >30× and 45,245 SNVs or small indel variants were identified. After filtration by the quality and population frequency, the number of variants affecting the coding sequence was reduced to 467 variants in 451 genes in the proband. Among the 451 genes carrying variation(s), there were seven genes known to be DSD causing or DSD candidates (Table S1) [26,27,28,29], namely *DHCR7* (7-dehydrocholesterol reductase; 11q13.4), *DYNC2H1* (dynein cytoplasmic 2 heavy chain 1; 11q22.3), *FREM2* (FRAS1 related extracellular matrix 2; 13q13.3), *SPECC1L* (sperm antigen with calponin homology and coiled-coil domains 1 like; 22q11.23), *SPECC1L-ADORA2A* (SPECC1L-ADORA2A readthrough; 22q11.23), *BBS9* (Bardet-Biedl syndrome 9; 7p14.3) and *WT1*. However, variants in four genes (*DYNC2H1*, *SPECC1L-ADORA2A*, *SPECC1L*, *BBS9*) were predicted to be of uncertain significance or benign using Meta-SNP (Table S1). For that reason, we did not further consider their influence on the patient’s phenotype. The other two variants of *DHCR7* and *FREM2* genes were predicted to be pathogenic or likely pathogenic, respectively (Table S1). However, the phenotype of the patient did not fit the Smith–Lemli–Opitz syndrome (MIM 270400) or 46,XY Frasier Syndrome (MIM 136680), which are associated with *DHCR7* and *FREM2*, respectively. Moreover, the same variants were also detected in the healthy father or mother. However, we cannot rule out the modifier effect of those variants.

**Figure 1:**
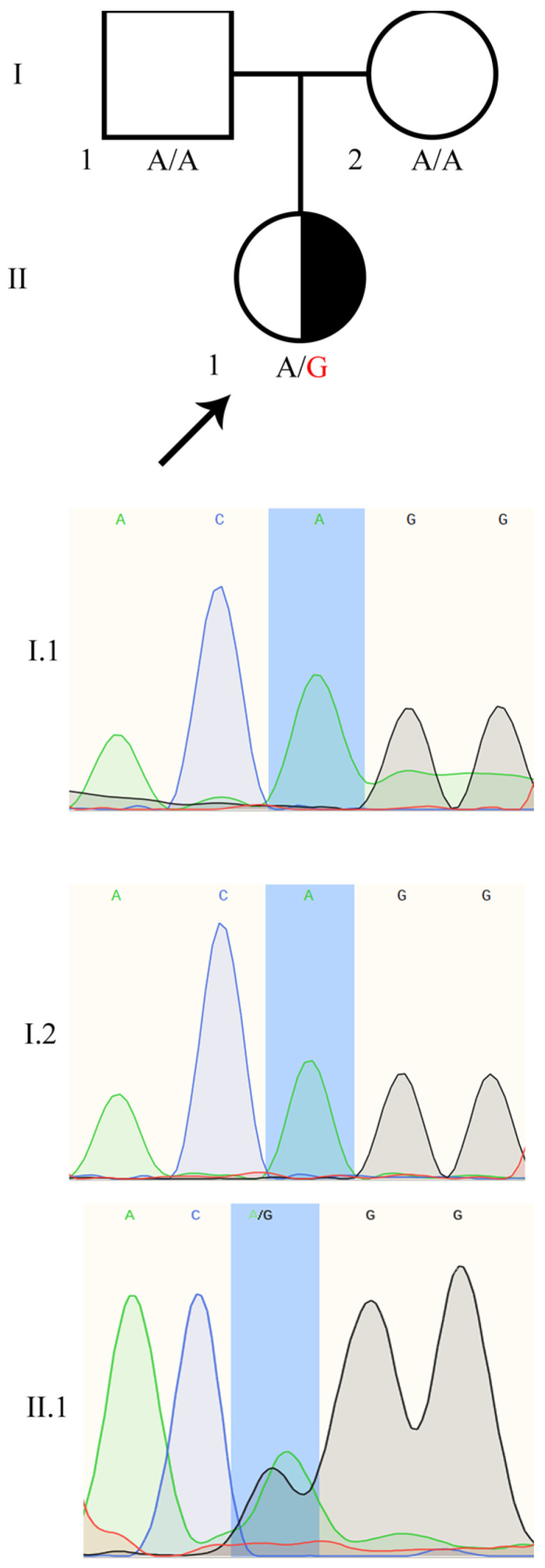
The confirmation of the *WT1*:c.1437 A>G substitution in the 46,XX TDSD/OTDSD patient. The family member analysis showed de novo occurrence of the mutation in the patient.

The only variant which drew our attention was a heterozygous transition in the DSD related *WT1* gene, NM_024426.6 c.1437 A>G (11:32413528 T/C GRCh37), not detected in the patient’s healthy parents. We confirmed the heterozygous status of the WT1:c.1437 A>G in the patient and the wild type status in both parents by Sanger sequencing (Figure 1). Importantly, the WT1:c.1437 A>G variant was not present in any SNV database. We registered the variant in ClinVar as ID: 981464.

### 2.3. The WT1:c.1437A>G Mutation Results in the Retention of Intron 9, Predicting Premature Translational Termination and Absence of Zinc Finger 4

**Figure 2:**
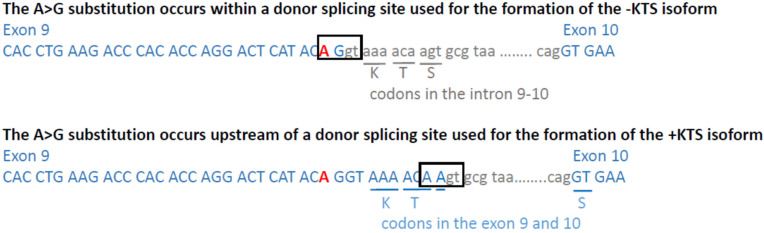
The localization of the WT1:c.1437A>G substitution in the exon 9 in relation to alternative donor splice sites. Two alternative donor splice sites are marked by rectangles. The upper and lower panels show splice sites used for the formation of the −KTS and +KTS isoforms, respectively. KTS codons in the +KTS isoform are underlined by blue lines, while nucleotides corresponding to them in the −KTS isoform are underlined by grey lines.

The WT1:c.1437A>G substitution identified in this study is localized at the end of exon 9 within the consensus sequence of the upstream alternative donor splice site which is normally utilised to produce the −KTS isoform (Figure 2). However, the Human Splicing Finder (HSF) [30] predicts that the substitution affects splicing from both alternative donor splicing sites. Moreover, according to HSF, the WT1:c.1437A>G substitution impairs the recognition by the SRp40 splicing factor 5 which plays a role in constitutive splicing and selection of alternative splice sites [31]. Thus, based on computational predictions, we expected the WT1:c.1437A>G mutation to result in the retention of intron 9.

**Figure 3:**
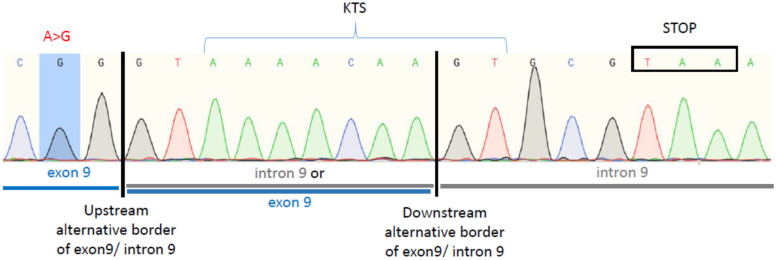
Confirmation of the *WT1* intron 9 retention in mRNA of the 46,XX TDSD/OTDSD patient carrying the WT1:c.1437A>G substitution. Sequencing of cloned RT-RCR product obtained from the cDNA of the patient shows presence of intron 9 sequence following exon 9 which contains the A>G substitution in the upstream alternative donor splice site. Exon 9 is marked in blue, intron 9 is marked in gray, the sequence which belongs to exon 9 or intron 9 depending on which alternative donor splicing site is used is marked in gray and blue.

**Figure 4:**
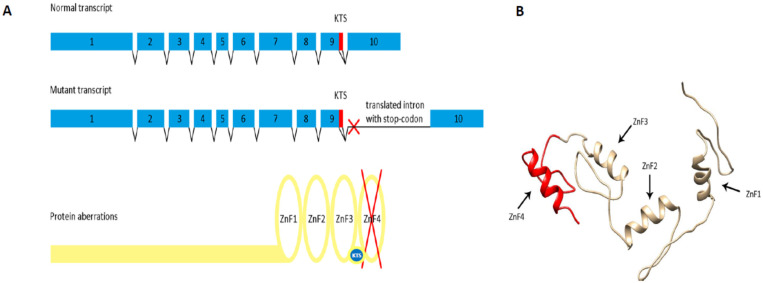
Aberrations of *WT1* transcript and protein as a result of WT1:c.1437A>G substitution. (**A**) The upper panel represents a wild type *WT1* alternative transcripts with KTS encoding sequence (marked with red) within the exon 9 or within the intron 9 depending on an isoform. The middle panel represents a mutated transcript containing retained intron 9 with STOP codon which is indicated as a red cross. The lower panel represents an aberrant *WT1* +KTS protein lacking Zinc Finger 4 encoded by the mutated transcript (**B**) *WT1* protein zinc fingers. Lost part of the DNA/RNA binding domain is marked with red color.

Further we investigated whether intron 9 is indeed retained in the patient carrying the WT1:c.1437A>G mutation. To that end, RT-PCR was performed using the patient’s cDNA and primers designed to produce a PCR product exclusively if intron 9 is retained in mature mRNA (Figure S2). The RT-PCR product was cloned into a plasmid and sequenced. Sequencing confirmed the presence of intron 9 downstream of the mutated exon 9, proving that the mutation indeed caused the intron 9 retention (Figure 3). Moreover, the intron contains a STOP codon located six nucleotides downstream of the KTS encoding triplets (Figure 3). In summary, if the erroneous mRNA is translated, the mutated allele encodes only +KTS protein lacking ZnF4 (+KTS/−ZnF4) (Figure 4) and no −KTS isoform.

## 3. Discussion

We discovered a WT1:c.1437A>G transition in a patient with 46,XX TDSD/OTDSD. To the best of our knowledge, this variant has never been described before, nor has it been found in any large genomic screens. In an attempt to predict the level of pathogenicity for the WT1:c.1437A>G variant, we followed the standards of the American College of Medical Genetics which are commonly used to report clinically relevant variants [32]. We assigned the WT1:c.1437A>G variant status as ‘pathogenic’ status because it meets the criteria PVS1 (null variant in a gene where loss of function is a known mechanism of disease), PM2 (the absence in global genomic screens) and PS2 (de novo variant). Therefore we have registered WT1:c.1437A>G in the ClinVar database.

The substitution occurred at the end of exon 9, in one of two alternative donor splice sites, the upstream one. We demonstrated that the mutation causes intron 9 retention and argue that the incorrect mRNA may encode the *WT1* +KTS protein lacking ZnF4. Sequence of an aberrant transcript indicates that the −KTS isoform is not produced at all by the mutated allele.

Until recently, *WT1* mutations were linked to 46,XY rather than 46,XX DSD. Specific pathogenic variants have earlier been found for two rare autosomal dominant diseases, Denys–Drash syndrome (MIM 194080) and Frasier syndrome (MIM 136680). These syndromes are characterized by renal anomalies and gonadal dysgenesis in 46,XY individuals, while 46,XX individuals with these syndromes typically have normal ovaries [24,33]. Denys–Drash syndrome is caused by mutations in exon 8 or 9 (encoding ZnF2 or ZnF3, respectively), believed to reduce the *WT1* protein’s ability concerning DNA binding [34]. In turn, Frasier syndrome is caused by mutations in the downstream alternative donor splice site within intron 9 leading to the loss of the +KTS isoform [24,25], which is required for male sex determination [23].

During the last two years, six distinct *WT1* mutations have been described in 46,XX TDSD/OTDSD patients [12,13] with phenotypes completely different from those associated with earlier reported *WT1* mutations (46,XY gonadal dysgenesis in Denys–Drash or Frasier syndrome). Interestingly, all the 46,XX TDSD/OTDSD associated mutations affect the ZnF4 [12,13]. The mutation WT1:c.1437A>G that we present here, also affecting ZnF4, is the seventh variant among those described in 46,XX TDSD/OTDSD. The *WT1* mutations described so far in 46,XX TDSD/OTDSD can be classified into three groups: (1) Missense mutations in exon 10 encoding ZnF4 (p.Lys491Glu, p.Arg495Gln, p.Arg495Gly) [12]; (2) deletions at the beginning of exon 10, resulting in a frameshift, premature stop codon occurrence and predicted ZnF4 loss (p.Pro481Leufs*15, p.Arg485Glyfs*14) [12,13]; (3) substitutions within splicing sites causing (our study) or expecting retention of intron 9 and formation of only +KTS isoform lacking ZnF4: c.1437A>G (NM_024426.6; current study) and c.1448-1G>A (NM_024426.6) described as 1433-1G>A by Eozenou et al. [12]. Since all the *WT1* mutations in 46,XX TDSD/OTDSD are localized within exon 10 encoding ZnF4 or within splicing sites involved in intron 9 splicing, those regions can be considered as a hot spot for 46,XX TDSD/OTDSD causative mutations. We noticed that ZnF4 is highly conserved from amphibian to human (Table S2) suggesting an essential role of this domain. However, the function of ZnF4 in the +KTS and −KTS isoforms was shown to be different. In the −KTS isoform, ZnF4 is required for DNA binding stabilization [35,36], while in +KTS, ZnF4 is important for interaction with RNA [37].

A substantial question that should be raised here is: Which molecular mechanism(s) was (were) disturbed by *WT1* mutations in 46,XY TDSD/OTDSD patients? The missense mutations (group 1) and frameshift mutations (group 2) presumably affect both +KTS and −KTS isoforms. Eozenou et al. demonstrated that point mutations p.Lys491Glu and p.Arg495Gly (same as 46,XX TDSD/OTDSD patients have), as well as one artificial mutation causing a loss of ZnF4 (p.Arg495*), enhance binding of *WT1* +KTS to Catenin beta 1 [12]. Since Catenin beta 1 is known to be a pro-ovarian factor [38], these results strongly suggest that point mutations within ZnF4 as well as a mutation causing lack of ZnF4 (p.Arg495*) are, in fact, gain-of function mutations leading to Catenin beta 1 sequestration, allowing for upregulation of pro-testicular pathways. Indeed, Eozenou et al. showed that *WT1* p.Arg495Gly mutation upregulates *SOX9*, *NR5A1 and DMRT1* associated with testicular Sertoli cell formation [12].

The splicing site mutations (group 3), including the one described in this work, represent a group believed to cause synthesis of truncated *WT1* +KTS protein lacking ZnF4, accompanied by the absence of −KTS isoform production from the mutated allele. The *WT1* truncated protein is expected to disturb formation of functional *WT1* homodimers which normally act as transcriptional factor [22,39]. In the case of our patient, where both normal and mutant proteins are expected to be produced, approximately 75% of all *WT1* dimers are going to be aberrant (25% of mutant/mutant dimers, 50% of mutant/wild-type dimers, 25% of wild-type/wild-type dimers). A scenario like this drastically reduces the possibility of wild-type protein performing proper molecular functions. However the proportion of aberrant *WT1* dimers may be changed if truncated *WT1* protein molecules undergo inactivation by oligomerization (since formation of complexes consisting of truncated and full-length *WT1* molecules was shown [40]). In each scenario, non-productive complexes (dimers or oligomers) decrease the amount of functional wild type *WT1* in dominant-negative mode [40] and therefore could change WT1-regulated gene expression, including genes critical for sex development. Absence of ZnF4 in the +KTS aberrant protein may as well increase an interaction with Catenin beta 1 (like it was shown for artificial mutant lacking ZnF4 [13]) leading to Catenin beta 1 sequestration and prevention of its pro-ovarian/anti-testicular function. Furthermore, because it has been established that +KTS is required for testicular determination, we propose that disruption of the +KTS/−KTS ratio (which is predicted) may contribute to the 46,XX TDSD/OTDSD phenotype as a modifier factor in addition to loss of ZnF4 sequence.

## 4. Materials and Methods

### 4.1. Patient Description

A patient of Ukrainian origin (UKR29) was born after the first normal pregnancy (39 week of gestation) from a healthy 27 year old mother and 32 year old father. At birth, the child was registered as a male. Birth weight was 3500 g and length was 53 cm. At the age of two months, the patient was examined due to hypospadias and bilateral cryptorchidism. At that time hormonal analysis was performed. At the age of 14 months a comprehensive examination, such as karyotyping, urological examination (including gonadal and pelvic ultrasound and MRI investigation) and hormonal analysis (including testosterone synthesis stimulation test) were performed. The patient’s psychological development was normal. Neither signs of Wilms’ Tumour nor renal anomalies were found in the patient.

Informed consent was obtained from the patient’s parents. Ethical approval for this study was obtained from the Committee on Bioethics of the Institute of Molecular Biology and Genetics of National Academy of Sciences of Ukraine, protocol No. 2 (30 April 2013).

### 4.2. Hormonal Analysis

hCG test was performed according to a published protocol [41]. hCG (Roche Diagnostics, Germany, Mannheim) was administered at 1500 IU for 3 days. The study of total testosterone in the blood was performed before the 1st hCG injection and 24 h after 3rd injection. The test is considered positive if the testosterone level is more than doubled compared to the basal level. Serum levels of free Testosterone (fT), LH, FSH and E2 were quantified by electrochemiluminescence immunoassay technology on Cobas E411 (Roche Diagnostics, Risch-Rotkreuz, Switzerland). The level of 17-OHP was quantified on Tecan SunRise (Tecan’s Magellan, Männedorf, Switzerland) using commercial kit (NovaTec, Dietzenbach, Germany). Elecsys Testosterone II, Elecsys LH, Elecsys FSH and E2 kits were used according to the manufacturer’s instructions (Roche Diagnostics, Germany, Mannheim). The AMH level was determined by the chemiluminescence immunoassay using paramagnetic particles by DXI 800 (Beckman Coulter, Inc., Brea, CA USA). The DHT level was determined by enzyme-linked immunosorbent assay (Demeditec, Kiel, Germany).

### 4.3. Cytogenetic Studies

Cytogenetic studies were performed on peripheral blood lymphocytes (30 metaphase plates) using Nikon Eclipse Ci microscope (Nikon, Minato, Japan). FISH analysis was performed at 200 interphase nuclei using LUCIA Cytogenetics Software (Praha, Czech Republic) according to Cytogenetic Guidelines and Quality Assurance by European Cytogenetics Association (GTG-banding, FISH—probes CEP, LSI (Probes: Yp11.3—*SRY*; Yp11.1-q11.1—DYZ3; Yq12—DYZ1; CEP—DXZ1) (Abbott Molecular, Libertyville, IL, USA).

### 4.4. Whole Exome Sequencing (WES)

Genomic DNA from the blood samples of the proband and his parents was isolated by using the QIAmp DNA Kit (Qiagen, Hilden, Germany). Exome capture was performed on the DNA samples using the SureSelectXT Target Enrichment system for Illumina version B.2 (Agilent Inc^®^, Santa Clara, CA, USA). Paired-end libraries were prepared using TruSeq SBS Kit v3 (Illumina, San-Diego, CA, USA) and sequenced on an Illumina HiSeq 4000 system (Illumina, San-Diego, CA, USA). Raw sequencing was transformed into fastq files using the CASAVA v1.8.1 software (Illumina, San-Diego, CA, USA) and processed with DRAGEN Germline Pipeline v 2.3 (Edico Genome), which leverages Genome Analysis Tool Kit (GATK; https://gatk.broadinstitute.org/hc/en-us/articles/360045944831, last accessed 26 November 2021) and is harboured at the Illumina’s cloud-based resource BaseSpace. For proband and his parents, 100 to 150 million reads were processed, adapter trimmed, duplicate marked and aligned against the GRCh37/hg19 assembly of human genome using Smith–Waterman scoring algorithm. At the variant calling stage, the following filters were applied: [Variant confidence/Quality by depth] > 2.0, [Mapping quality] > 30.0, [Phred-scaled p-value for strand bias] < 60.0, [Mapping quality RankSum] > −12.5, [ReadPosRankSum] > −8.0. Variants were annotated and analysed in Variant Interpreter (Illumina, San-Diego, CA, USA) and VarSeq (Golden Helix, Boseman, MT, USA). Further filtering was performed by the variant quality (>500), genotyping quality (>80), read depth (>30), proportion of reads bearing the minor allele (>0.2), population frequency (<0.01).

### 4.5. The WT1:c.1437A>G Variant Validation by Sanger Sequencing

The variant WT1:c.1437A>G identified in the *WT1* gene by WES was validated using Sanger sequencing in the patient, as compared to his parents. The fragment containing exon 9 and adjacent parts of intron 8 and 9 was amplified using the gDNA template of the patient or his parents by primers F:GACCTTCTCTGTCCATTTAG and R:CTCCTTCTCTGTATTTCCAC. PCR was done with the use of 5xHOT FIREPol ^®^ Blend Master Mix (Solis BioDyne, Tartu, Estonia) according to the manufacturer’s instructions. The 286 bp amplicon was sequenced in both directions using the above primers, in separate reactions. Sequencing was performed using BigDye ^®^ Terminator Kits (Thermo Fisher Scientific, Waltham, MA, USA) on 3130 Genetic Analyzer (Applied Biosystems, Thermo Fisher Scientific, Waltham, MA, USA). Chromatograms were analysed and converted from.ab1 format to fasta format using the open source SnapGene 4.3.11 software (GSL Biotech LLC, San-Diego, CA, USA).

### 4.6. Bioinformatic Resources

The data of the Genome Aggregation Database (gnomAD) [42], the 1000 genomes project [43], the exome sequencing project (Exome Variant Server, https://evs.gs.washington.edu/EVS/) and the Exome Aggregation Consortium (ExAC) Browser [44] were used to check the WT1:c.1437A>G variant allele frequency. Public medical genetic databases OMIM (https://www.omim.org/, last accessed 26 November 2021), ClinVar (https://www.ncbi.nlm.nih.gov/clinvar/, last accessed 26 November 2021) and HGVD (https://www.hgvd.genome.med.kyoto-u.ac.jp/, last accessed 26 November 2021) were used for the WT1:c.1437A>G variant pathogenicity evaluation. Human Splicing Finder tool [30] was used for the WT1:c.1437A>G variant impact assessment.

### 4.7. Influence of the WT1:c.1437A>G Variant on Splicing

Total RNA was extracted from 250 μL of whole blood using Tri Reagent BD (Sigma-Aldrich, Merck, Darmstadt, Germany) together with dsDNase pre-treatment (Thermo Fisher Scientific, USA). The amount and purity of obtained RNA were evaluated with the NanoDrop spectrophotometer (Saveen Verner, Limhamn, Sweden). cDNA was synthesized using the Maxima H Minus First Strand cDNA Synthesis Kit (Thermo Fisher Scientific, Waltham, MA, USA). Lack of gDNA in the sample was ascertained by RT-PCR using a pair of primers (F: GGACTTCGAGCAAGAGAT and R: AGCACTGTGTTGGCGTAC) complementary for an exon and a following intron of the housekeeping *ACTB* gene (Figure S1). Retention of *WT1* intron 9 was investigated using RT-PCR with primers F:GTGTGAAACCATTCCAGTGT complementary to sequence in exon 9 and R:CCCTCTCATCACAATTTCAT complementary to intronic 9 sequence (Figure S2). The 168 bp amplicon was extracted and purified from agarose gel using Silica Bead DNA Gel Extraction Kit (Thermo Fisher Scientific, Waltham, MA, USA) and cloned into pJET1.2 vector (CloneJET PCR Cloning Kit, Thermo Fisher Scientific, Waltham, MA, USA). Insertion was verified by restriction analysis using XhoI and XbaI (NEB, Ipswich, MA, USA). Clones carrying correct size insert were sequenced from the pJET1.2 forward and reverse sequencing primers (Applied Biosystems 3130xl, Thermo Fisher Scientific, Waltham, MA, USA).

### 4.8. D Protein Modelling

Modelling of *WT1* +KTS DNA/RNA binding domain form was performed using the I-Tasser server [45] with PDB ID: 6BLW assigned as a template.

### 4.9. Accession Numbers

Accession number of *Homo sapiens WT1* mRNAs is NM_024426.6. UniProt IDs of vertebrate *WT1* proteins used are: *Homo sapiens* P19544, *Sus scrofa* O62651, *Rattus norvegicus* P49952, *Mus musculus* P22561, *Sminthopsis macroura* P49953, *Gallus gallus* Q9I8A0, *Alligtor mississippiensis* P50902, *Xenopus laevis* B7ZSG3, *Rugosa rugosa* A9CSE6.

## 5. Conclusions

A novel de novo splice site *WT1* gene mutation, WT1:c.1437A>G, identified in a 46,XX TDSD/OTDSD patient causes retention of the intron 9. If the aberrant transcript is translated, exclusively a truncated *WT1* +KTS protein lacking ZnF4 is expressed from the mutated allele. This mutation may act as a double-edge knife: (1) On the one hand, it impairs *WT1* +KTS functions related to nucleic acids binding, and (2) on the other hand, it disturbs +KTS/−KTS ratio with +KTS excess, which may additionally contribute to the 46,XX TDSD/OTDSD phenotype as a modifier factor.

## Figures and Tables

**Table 1:** Values of free testosterone (fT) baseline and after human chorionic gonadotropin (hCG) stimulation, dihydrotestosterone (DHT), anti-Müllerian hormone (AMH), estradiol (E2), luteinizing hormone (LH), follicle-stimulating hormone (FSH) and 17-hydroxyprogesterone (17-OHP) of the 46,XX TDSD/OTDSD patient.

Hormone, Unit		Age 2 Months	Age 2 Months Reference Values	Age 14 Months	Age 14 Months Reference Values
fT, ng/dL	Baseline			0.001	♀ < 0.5 ♂ < 0.5
	24 h after 3-days hCG stimulation			0.321	
DHT, ng/dL				1.186	♀ < 3 ♂ < 3
AMH, ng/mL				10.56	♀ 0.17–8.9 ♂ 3.8–159.8
E2, pg/mL		0.23	♀ < 15 ♂ < 15		
LH, mIU/mL		11.19	♀ 0.02–7.0 ♂ 0.02–7.0	<0.1	♀ 0.02–0.3 ♂ 0.02–0.3
FSH, mIU/mL		6.43	♀ 0.24–14.2 ♂ 0.16–4.1	1.01	♀ 0.3–11.1 ♂1.0–4.2
17-OHP, ng/mL				0.43	♀ 0.1–4.0 ♂ 0.1–4.0

## Data Availability

Data available on request due to ethical restrictions. The data presented in this study are available on request from the corresponding author.

## Supplementary Materials

The following are available online at https://www.mdpi.com/article/10.3390/biology10121248/s1, Figure S1: *ACTB* RT-PCR demonstrates that cDNA sample obtained from the studied patient blood is gDNA-free, Figure S2: Pair of primers used for aberrant transcript detection in cDNA of the 46,XX TDSD/OTDSD patient carrying the WT1:c.1437A>G substitution, Table S1: List of heterozygous variants in autosomal genes involved in sexual development identified in the 46,XX TDSD/OTDSD patient, Table S2: Results of multiple alignment of *WT1* zinc finger 4 amino acid sequence.
