# Supplementary material for: A Novel WT1 Mutation Identified in a 46,XX Testicular/Ovotesticular DSD Patient Results in the Retention of Intron 9
Source: Biology (Basel). 2021 Nov 30;10(12):1248. doi: 10.3390/biology10121248 (PMC8698877; doi:10.3390/biology10121248)
Supplement: Supplementary file 1 [file biology-10-01248-s001.zip › biology-1471339-supplementary.pdf]

Supplementary Table S1. List of heterozygous variants in autosomal genes involved in sexual development identified in the 46,XX (O)TDSD patient.

Gene/ protein	Associated recessive diseases	Related pathway	SNP ID	Frequency (gnomAD)	DNA change	Protein change	Mother	Father	Meta-SNP
DHCR7<br>Delta-7-Dehydrocholesterol Reductase	Smith-Lemli-Opitz Syndrome and Holoprosencephaly, vitamin D deficiency	Terpenoid backbone biosynthesis and Metabolism	rs138659167	0.011822	c.964-1G>C	Splice acceptor	-	Het	Suspected pathogenic
FREM2<br>FRAS1-related extracellular matrix protein 2	Fraser Syndrome 2 (characterized by ambiguous genitalia in karyotypic males)	Extracellular matrix-receptor interaction	rs143044921	0.009505	c.4031G>A	p.(Arg1344His)	Het	-	Likely pathogenic
DYNC2H1<br>Dynein Cytoplasmic 2 Heavy Chain 1	Short-Rib Thoracic Dysplasia 3 With Or Without Polydactyly	Signalling by GPCR and Organelle biogenesis and maintenance	rs372204188	0.005326	c.10282A>G	p.(Lys3428Glu)	-	Het	Variant of uncertain significance
SPECC1L-ADORA2A<br>Sperm Antigen with Calponin Homology and Coiled-Coil Domains 1-Like and Adenosine A2a Receptor readthrough		Naturally occurring readthrough transcription between the neighbouring genes on chromosome 22, and is unlikely to produce a protein product	rs201337978	0.002122	Non-coding exon	n.778G>A	-	Het	Variant of uncertain significance
SPECC1L<br>Sperm Antigen with Calponin Homology and Coiled-Coil Domains 1-Like		Sperm antigen with calponin homology and coiled-coil domains 1-like, involved in cytokinesis and spindle organization. May play a role in actin cytoskeleton organization and microtubule stabilization and hence required for proper cell adhesion and migration			c.470G>A	p.(Arg157Gln)			
BBS9<br>Bardet-Biedl Syndrome 9	Bardet-Biedl syndrome 9	The exact function of this gene product has not yet been determined, thought to function as a coat complex required for sorting of specific membrane proteins to the primary cilia.	rs138072724	0.013056	c.1280C>T	p.(Ala427Val)	Het	-	Benign
WT1<br>Wilms' Tumor 1		Plays an essential role for development of the urogenital system.			c.1422A>G	p.(Thr474=)	-	-	Likely Benign

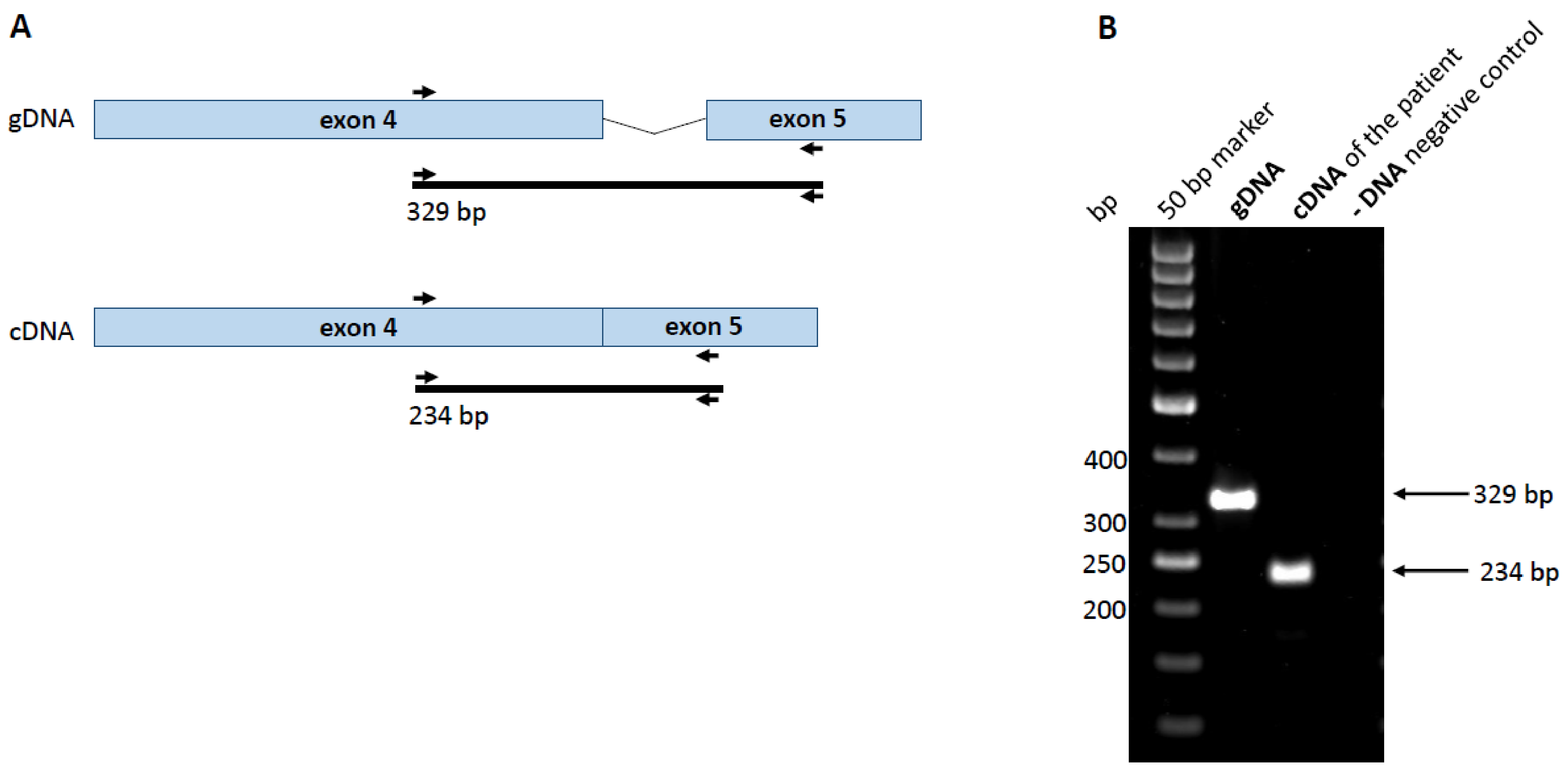

Supplementary Figure S1. *ACTB* RT-PCR showing that cDNA sample obtained from the studied patient blood is gDNA-free. **A:** Strategy for cDNA analysis. For amplification, a primer complementary for exon 4 (F:GGACTTCGAGCAAGAGAT) and exon 5 (R:AGCACTGTGTTGGCGTAC) of *ACTB* transcript ENST00000646664.1 were used. The upper panel represents amplification of a fragment of genomic *ACTB* (329 bp) including 95 bp intron. The lower panel represents amplification of a fragment of *ACTB* cDNA (234 bp) lacking the 95 bp intron sequence. Positions of the primers used for amplification are marked with the arrows. **B:** Amplification results: amplification characteristic for gDNA (329 bp amplicon) did not occur in the cDNA of the patient indicating that the cDNA sample is gDNA free.

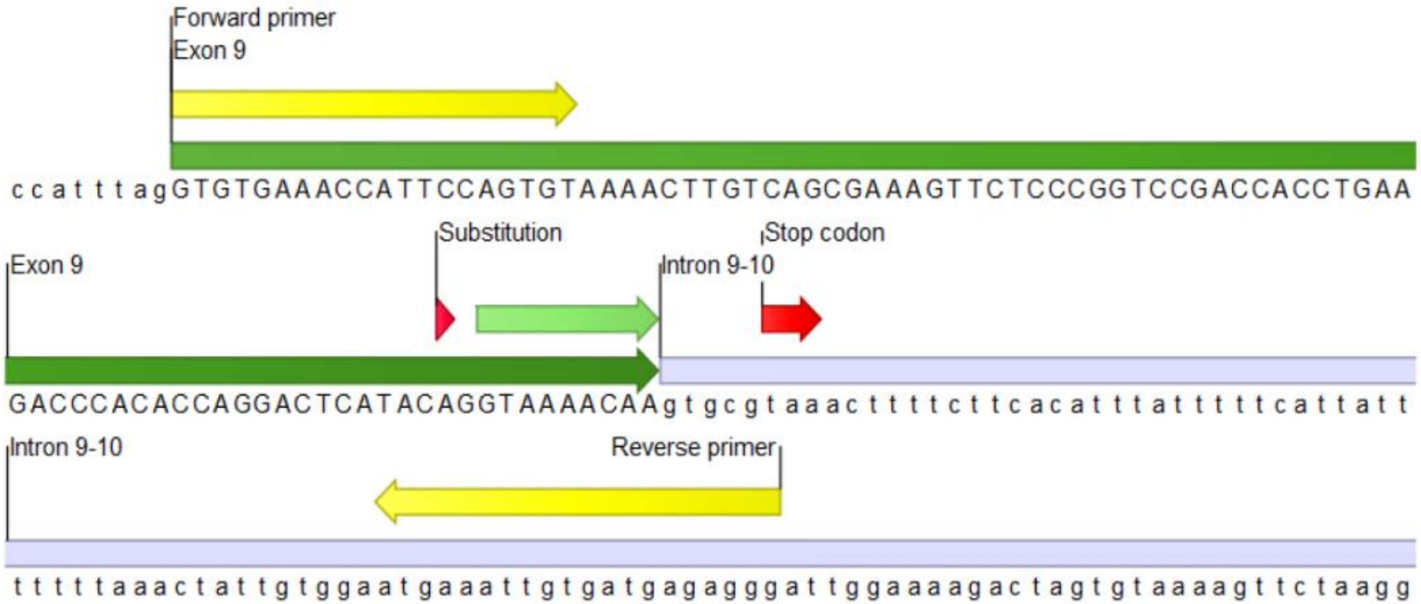

Supplementary Figure S2. Pair of primers used for aberrant transcript detection in cDNA of the 46,XX (O)TDSD patient carrying the WT1:c.1437A>G substitution. Green arrow represents fragment of RNA, that is either being retained (+KTS isoform), or spliced out (-KTS isoform).

Supplementary Table S2. Results of multiple alignment of zinc finger 4 amino acid sequence. Results are displayed in percentage of matches. Highlighted in red are results below 95%. All sequences match ≥90% of the sequence.

	Homo sapiens	*Sus scrofa*	*Rattus norvegicus*	*Mus musculus*	*Sminthopsis macroura*	*Gallus gallus*	*Alligator mississippiensis*	*Xenopus leavis*	*Rugosa rugosa*
Homo sapiens									
Sus scrofa	97,5%								
Rattus norvegicus	92,5%	90,0%							
Mus musculus	92,5%	90,0%	100,0%						
Sminthopsis macroura	97,5%	95,0%	90,0%	90,0%					
Gallus gallus	100,0%	97,5%	92,5%	92,5%	97,5%				
Alligator mississippiensis	100,0%	97,5%	92,5%	92,5%	97,5%	100,0%			
Xenopus leavis	100,0%	97,5%	92,5%	92,5%	97,5%	100,0%	100,0%		
Rugosa rugosa	97,5%	95,0%	92,5%	92,5%	95,0%	97,5%	97,5%	97,5%	
